# State of the art on the separation and purification of proteins by magnetic nanoparticles

**DOI:** 10.1186/s12951-023-02123-7

**Published:** 2023-10-04

**Authors:** Thanh-Do Le, Itthanan Suttikhana, Tolulope Joshua Ashaolu

**Affiliations:** 1 Institute for Global Health Innovations, Faculty of Medicine, Duy Tan University, Da Nang, 550000 Vietnam; 2 Department of Multifunctional Agriculture, Faculty of Agriculture and Technology, University of South Bohemia, České Budějovice, Czech Republic

**Keywords:** Protein separation and purification, Surface modification, Magnetic nanoparticles, IMAC, Poly-his pre-tagged proteins

## Abstract

The need for excellent, affordable, rapid, reusable and biocompatible protein purification techniques is justified based on the roles of proteins as key biomacromolecules. Magnetic nanomaterials nowadays have become the subject of discussion in proteomics, drug delivery, and gene sensing due to their various abilities including rapid separation, superparamagnetism, and biocompatibility. These nanomaterials also referred to as magnetic nanoparticles (MNPs) serve as excellent options for traditional protein separation and analytical methods because they have a larger surface area per volume. From ionic metals to carbon-based materials, MNPs are easily functionalized by modifying their surface to precisely recognize and bind proteins. This review excavates state-of-the-art MNPs and their functionalizing agents, as efficient protein separation and purification techniques, including ionic metals, polymers, biomolecules, antibodies, and graphene. The MNPs could be reused and efficaciously manipulated with these nanomaterials leading to highly improved efficiency, adsorption, desorption, and purity rate. We also discuss the binding and selectivity parameters of the MNPs, as well as their future outlook. It is concluded that parameters like charge, size, core–shell, lipophilicity, lipophobicity, and surface energy of the MNPs are crucial when considering protein selectivity, chelation, separation, and purity.

**Graphical abstract:**

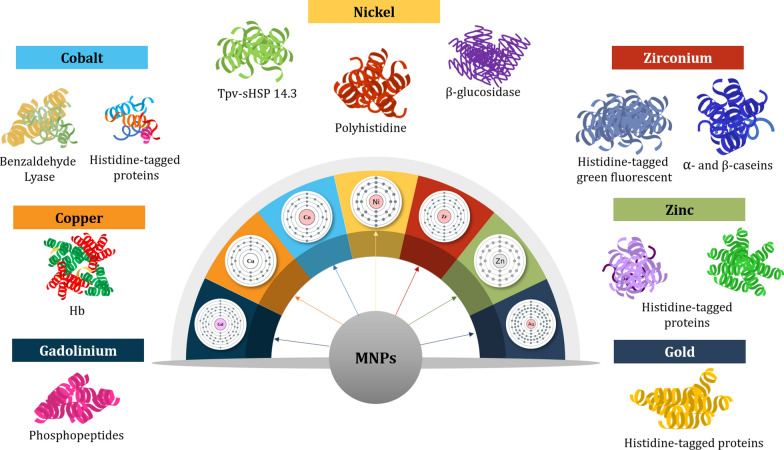

## Introduction to protein analytical techniques

Proteins are biomacromolecules that play a crucial role in living organisms. This makes their isolation, purification, and characterization important. In fact, several diseases are diagnosed early based on the characterization of trace proteins in human blood [71], and the techniques employed usually target antigens, antibodies, metals, electrostatic adsorption, hydrophilic and hydrophobic activities, and their interactions [17, 54, 128]. Over time, the methods have been improved and enhanced, leading to the rediscovery of novel materials meant to enrich and purify micro-molecules and more complex biomacromolecules [16].

In non-biological samples, protein identification techniques include sequencing, Edman degradation, gel electrophoresis (polyacrylamide, two dimensional, isoelectric focusing), isotope labelling (enzymatic, an isotope-coded affinity tag, stable-isotope labelling in cell culture), matrix-assisted laser desorption/ionization-time-of-flight mass spectrometry (MALDI-TOF), mass spectrometry [MS-liquid chromatography–mass spectrometry (LC–MS), and tandem mass spectrometry (TANDEM MS)], enzyme-linked immunosorbent assay [ELISA (competitive, indirect, reverse, sandwich)], and immunohistochemistry [14]. Structural analyses of the proteins are conducted using electron microscopy, circular dichroism, nuclear magnetic resonance (NMR) spectroscopy, and x-ray crystallography methods, while protein purification techniques commonly employed are based on chromatography i.e., column, size-exclusion (gel-filtration), ion-exchange, affinity, and reverse-phase high-performance liquid chromatography. Characterization and quantification analyses of proteins often employ the Kjeldahl, Warburg (UV absorption), Coomassie-blue (Bradford), biuret, and Folin–Lowry methods, and sometimes MS for more precision [14].

Proteins and their shorter fragments known as peptides could be in bioproduct mixtures containing colloids and cellular impurities, and as such various techniques are used to remove them. They include isolation and purification techniques like electrophoresis, flocculation, centrifugation, high-performance liquid chromatography (HPLC), ultrafiltration, fast protein liquid chromatography (FPLC), liquid–liquid extraction, microfiltration, adsorption, and precipitation [28, 64, 74]. When these techniques are used, a considerable number of targeted products are lost. Also, the purification and recovery of proteins and other biomolecules from biological samples and various mixtures could be very expensive, cumbersome, and time-consuming, not to mention the potential human errors [109]. Therefore, novel separation and purification techniques that overcome these limitations are warranted.

Magnetic nanomaterials nowadays have become the subject of discussion in proteomics, drug delivery, and gene sensing due to their various abilities including rapid separation, superparamagnetism, and biocompatibility [9, 110, 133]. Magnetic materials serve as excellent options for regular protein separation and analytical methods because they are more powerful and possess a high surface area-to-volume ratio, which makes it easier to functionalize them at nanoscale ranges. For instance, polymer brush-modified magnetic nanoparticles and core–shell micro/nanoflowers are magnetic materials-based techniques designed to overcome the expensive, cumbersome, and rather inefficient separation methods used for most antibodies’ purification, which are typically based on protein A or G affinity chromatography [83]. The conventional chromatographic method is plagued with a plethora of insoluble lipoproteins, lipids, and other impurities on a microscale, which can develop clogs in the expensive column and lead to delays in processing time or back pressure, among other issues [55]. Presently, there are protein A-coated magnetic particles in the market primarily used in the purification of antibodies. However, novel and progressive downstream techniques beyond the conventional approaches that apply to processing biological molecules like amino acids, peptides, hormones, antibodies, immunoglobulins, growth factors, DNAs, proteins, and recombinant proteins in trace amounts are warranted.

## Magnetic nanoparticles (MNPs)

Novel nanomaterials are developed continuously to have enhanced physicochemical properties above conventional materials. Among these nanomaterials are the magnetic nanoparticles (MNPs). The MNPs can be manipulated with magnetic fields since they have active nanostructures and functionality that is based on their magnetic and chemical components [3, 33]. MNPs are great separation and purification materials that are famous for their selectivity based on molecular adsorption to magnetic particles, cost-effectiveness, and environmental sustainability [92]. When compared to conventional techniques like electrophoresis or chromatography, pre-treatment of samples is not required, making MNPs very efficient and with a high operational speed and accuracy [33].

Certain polymers and metals bind super-fast to proteins, and when they are functionalized by MNPs, they become more efficient as separation and purification molecules that can be reused [112]. Other than their high recoverability by external magnets, MNPs also affect liquid structures and thus are incorporated in the procedures of aqueous extraction and purification, to create a functionally active surface to capture biomacromolecules like nucleic acids, bacteria and proteins [12, 115]. Since metals constitute the vast portion of MNPs, iron oxides (Fe_3_O_4_) are the commonest ones utilized to date, due to their capacity to form strong bonds with proteins via covalent, non-covalent, physical or biological interactive forces [103], [5], [98]. They can be used alone or with coupling agents to increase the efficacy of the MNPs’ functionalization potentials when separating and purifying proteins.

Apart from Fe_3_O_4_, α-Fe_2_O_3_ (hematite), γ-Fe_3_O_4_ (maghemite), SiO_2_, CoFe_2_O_4_, and NiFe_2_O_4_ are among the popular MNPs that have been documented in numerous reports [41, 75, 97, 121]. Industrially, the protocols for the use of these MNPs are easy to scale up [99]. However, some of them are toxic and not biocompatible despite their high levels of magneticity. To this end, oxidization of MNPs like ferrite (Fe_2_O_3_) showed some anti-toxicity promises, while Fe_3_O_4_/silicon dioxides are among the core–shell nanostructures that have been developed to likewise ameliorate this issue [77, 119]. Summarily, MNPs are in their early developmental stage despite being used in the separation of amino acid isomers, when compared to agelong and well-established techniques such as chromatography [22], [107].

## Separation and purification of proteins by MNPs

**Fig. 1:**
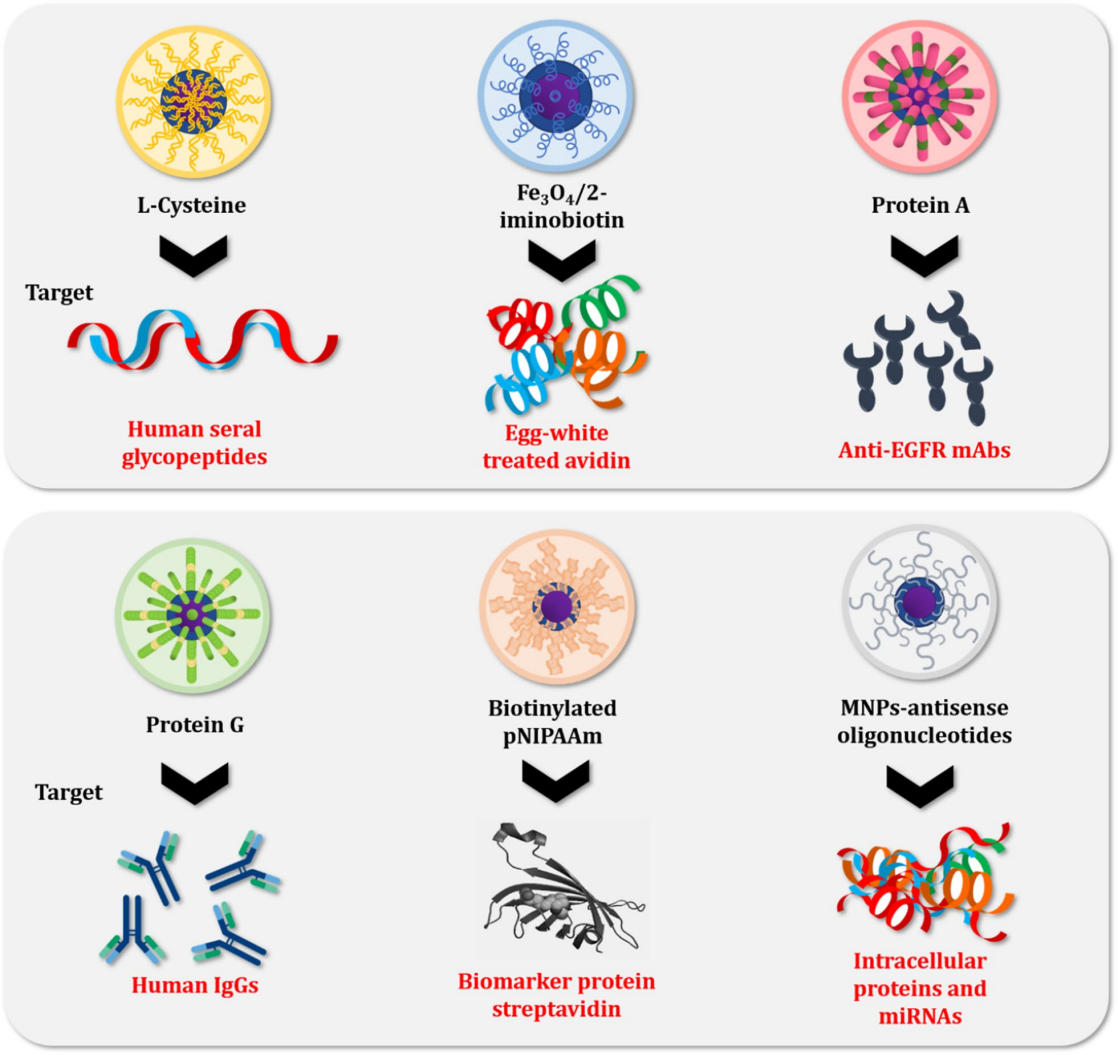
HYPERLINK "sps:id::fig1||locator::gr1||MediaObject::0" Functionalized MNPs with ligands like biomolecules increase the recognition and binding of proteins, resulting in magnetic separation and purification. Adapted from Eivazzadeh-Keihan et al. [33]

**Fig. 2:**
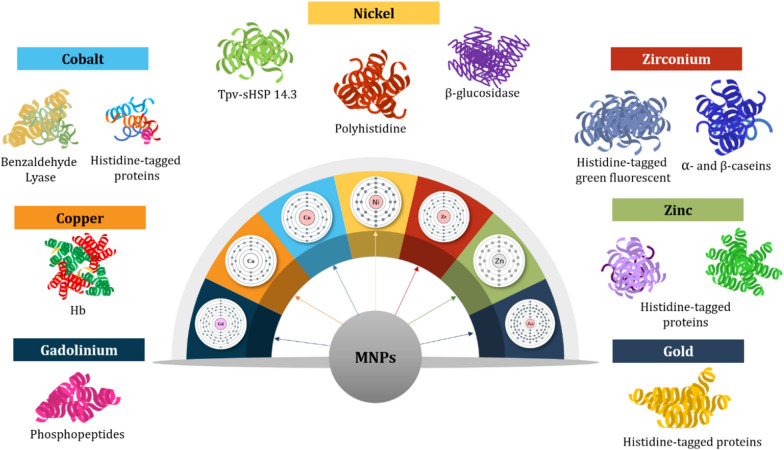
Functionalization of MNPs with ionic metals offers multifarious effective applications for separating and purifying proteins, particularly histidine-tagged proteins from biological samples

**Fig. 3:**
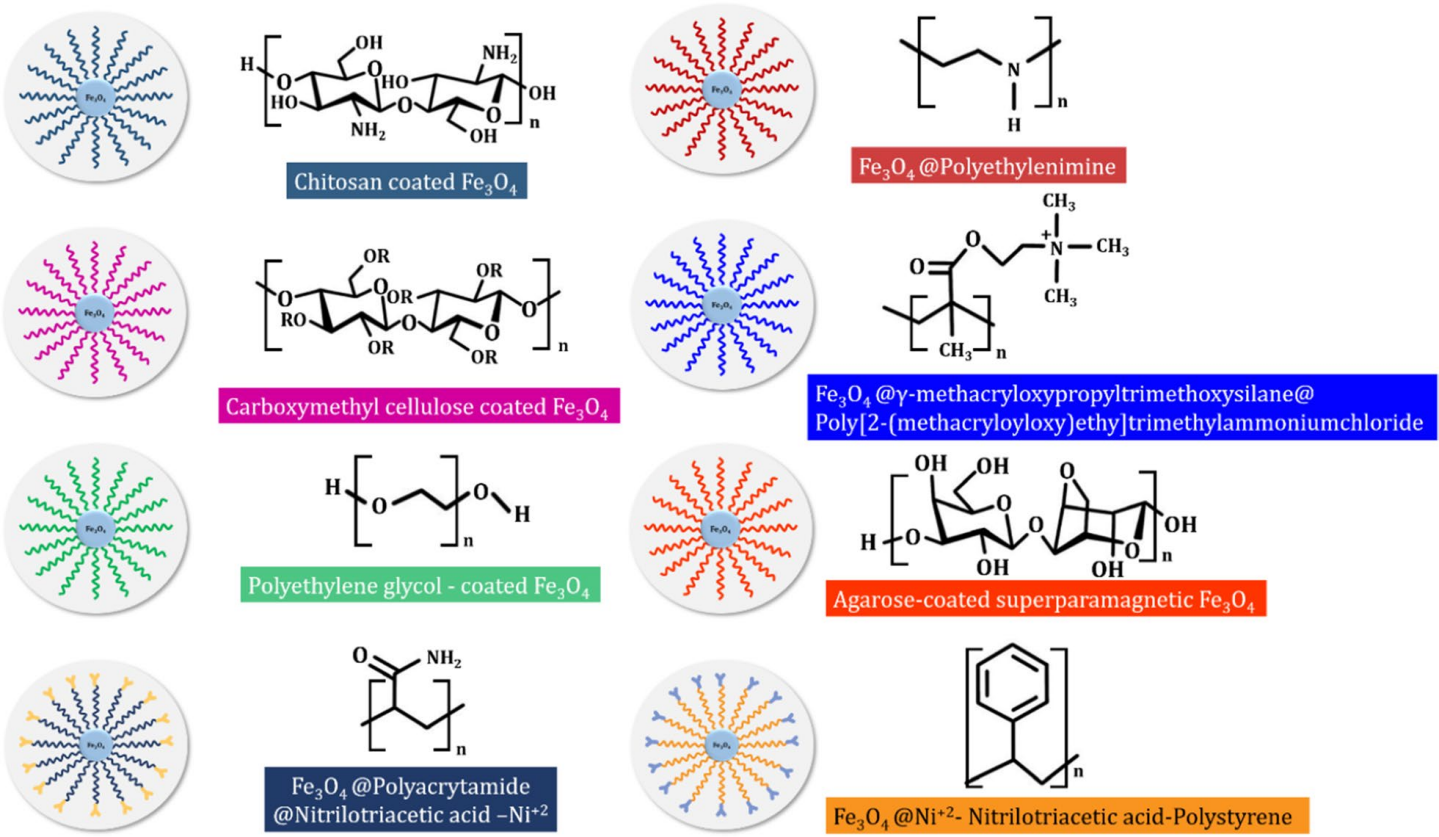
The complexation of polymers with MNPs serves as an excellent method of isolation and purification. *Adapted from* Eivazzadeh-Keihan et al. [33]

**Fig. 4:**
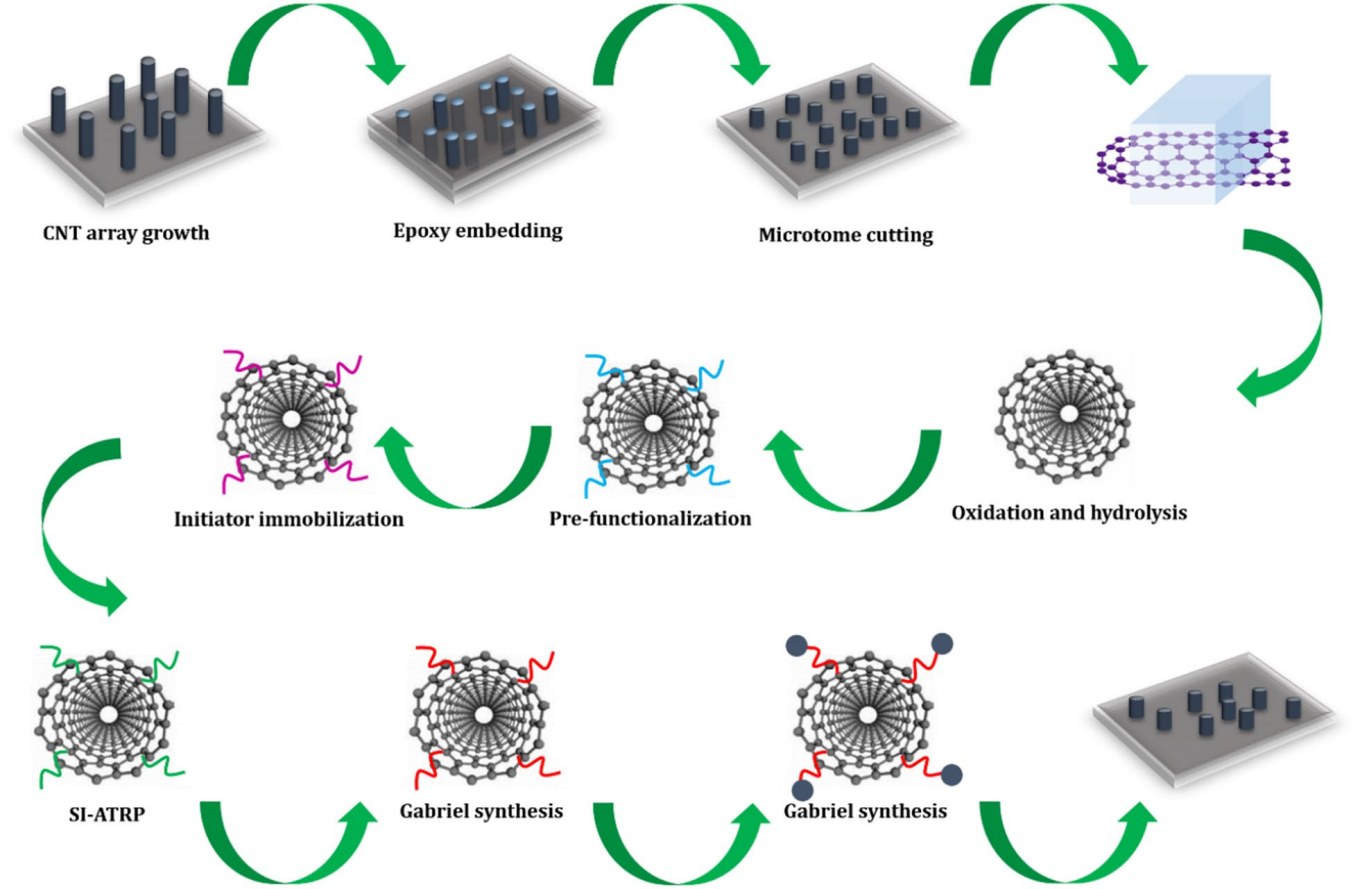
The preparation of *p*NIPAM-MAG-CNMs. A thermal-sensitive poly(*N*-isopropylacrylamide) (*p*NIPAM) is grafted with Fe_3_O_4_ nanoparticles onto the carbon nanotubes’ open ends using the surface-initiated atom transfer radical polymerization (SI-ATRP) method. The cavities of the nanotubes could transport both proteins and ions effectively and could be reversed in operation in a magnetic-thermal sensitive way to separate proteins of different sizes. *Adapted from* Cong et al. [26]

Various types of proteins and proteinaceous compounds are separated and purified at greater efficiency and accuracy with functionalized MNPs as compared to conventional techniques. This section describes the potential and usage of the commonest MNPs in this regard. The summaries are also presented in Tables 1, 2 and 3, with some schematic illustrations (Figs. 1, 2, 3, 4).

**Table 1:** Reported studies on the purification of proteins with biomolecules and functionalized MNPs

Composites of core–shell	Size (nm)	Technique of synthesis	Protein target	References
Covalent immobilization of protein A onto Glu activated Fe_3_O_4_@SiO_2_ MNPs (Fe_3_O_4_@SiO_2_ core–shell MNPs/proteinA)	~ 72	Solvothermal	Anti-EGFR monoclonal antibody (anti-EGFR mAb)	Hou et al. [53]
GNTA-SPION (3-glycidoxypropyl-trimet hoxy-silane Nα,Nα-bis(carboxymethyl)-l-lysine coupled superparamagnetic iron oxide nanoparticles)	6.5	Solvothermal	Recombinant proteins (protein A and α-lysozyme D1.3scFv antibody fragment from *Bacillus megaterium* cultivation)	Gädke et al. [44]
SD-SPION (spray-dried GNTA-SPION)	6.6			
GNTA-SPION (3-glycidoxypropyl-trimet hoxy-silane Nα,Nα-bis(carboxymethyl)-l-lysine coupled superparamagnetic iron oxide nanoparticles)	8–10	Non-aqueous sol–gel method	Histidine-tagged protein A (from *Bacillus megaterium*)	Gädke et al. [43]
Magnetic particles of azocasein-iron composite (mAzo)	–	Solvothermal	Trypsin (from tilapia fish crude extract)	Alves et al. [1]
Biofunctional magnetic nanoparticles bound to iminobiotin (Fe_3_O_4_/2-iminobiotin)	30	Solvothermal	Avidin (from treated egg-white solution)	Sun et al. [104]
Mesoporous silica coated magnetic microspheres with perfluorooctyl moieties on interior pore walls (Fe_3_O_4_@mSiO_2_-C_8_F_17_)	346	Surfactant templated sol–gel reaction	Fluorescent-labeled N-linked glycans	Zhao and Deng [141]
Glucose functionalized magnetic graphene hydrophilic nanocomposite (MagG/Au/Glu)	15	Solvothermal	Glycopeptides (from human serum)	Feng et al. [39]
Biotinylated poly(N-isopropylacrylamide) (pNIPAAm)- modified AuNPs mixed with pNIPAAm-coated iron oxide magnetic nanoparticles (mNPs) (pNIPAAm- AuNPs + pNIPAAm-mNPs)	pNIPAAm- AuNPs: 23 nm	UUsing laboratory syringe pumps	Biomarker protein streptavidin (in spiked human plasma)	Nash et al. [78]
	pNIPAAm-mNPs: 10 nm			
Bifunctional Au–Fe_3_O_4_ Nanoparticles (thiol modified Fe_3_O_4_ NPs mixed with Au NPs)	–	–	Arginine kinase (from cell lysate)	Bao et al. [6]
Magnetic nanoparticles (MNPs) of Fe_3_O_4_ immobilized with the ligand-binding domain (LBD) of estrogen receptor alpha (ERα) (LBD-ERα-immobilized MNPs)	142.4	Co-precipitation	Phytoestrogens (standard 17β-estradiol (E2)) in plant extracts (*Asparagus racemosus*)	Busayapongchai and Siri [13]
Magnetic glyconanoparticles (Supramolecular complexation between Fe_3_O_4_@Polyethylene Glycol@Adamantane and β-cyclodextrins conjugated with hepta-mannose (β-CD-(Man)7) and conjugated with poly(mannose) (β-CD-p(Man)280) (Fe_3_O_4_@PEG@AD + β-CD (Man)_7_/β-CD-p(Man)_280_)	22	Thermal decomposition	Concanavalin A (ConA) lectin (from a mixture of ConA and peanut agglutinin (PNA))	Oz et al. [82]
Magnetic nanoparticles functionalized with antisense oligonucleotides (Fe_3_O_4_@SiO_2_–COOH NPs)	14.6 ± 1.1	–	MicroRNAs (miRNAs) and intracellular proteins	Gessner et al. [46]
α-Glucosidase functionalized magnetic nanoparticles (αG-MNPs)	20	–	α-Glucosidase inhibitors (from *Dioscorea opposita* Thunb peel extract)	Chen et al. [21]

**Table 2:** Reported studies on the use of ionic metals functionalized with MNPs for protein purification

Technique of synthesis	Size (nm)		Functionalizing metal	Core material	Protein target	References
One-pot hydrothermal	140		Nickel	Iron oxide	Poly-histidine	Liu et al. [72]
–	80		Nickel	Iron oxide	Histidine-tagged proteins	Yao et al. [123]
Ti foils anodization	125–230		Fe_3_O_4_	TiO_2_	Histidine-tagged recombinant proteins	Kupcik et al. [62]
–	15		Nickel	Iron oxide	Enhanced green fluorescent protein	Wang et al. [114]
–	30		lanthanide elements	Iron oxide	Phosphopeptide	Zhai et al. [124]
–	220		Copper	Iron oxide	Bovine haemoglobin	Shi et al. [101]
–	220		Indium oxide	Iron oxide	Phosphopeptide	Jiang et al. [58]
Thermal plasma and chemical method involving co-precipitation of nitrate salts	28–32		Nickel	Iron oxide	Bovine serum albumin	Bhosale et al. [7]
Thermal plasma	240		Nickel–Zinc	Iron oxide	Histidine-tagged proteins	Feczkó et al. [37]
–	6–8		Nickel	Iron oxide	Histidine-tagged proteins	Sahu et al. [96]
Solvothermal Method	200		Nickel	Iron oxide	Histidine-tagged red fluorescent protein	Yang et al. [122]
Coprecipitation method	221		Titanium	Iron oxide	Phosphopeptide	Piovesana et al. [88]
Coprecipitation method	70		Nickel	Iron oxide	Histidine-tagged proteins	Rashid et al. [93]
High-temperature diol reduction method	5		Copper	CoFe_2_O_4_	Bovine haemoglobin	Li et al. [65]
	–		Nickel	Iron oxide	Pullulanases	Wang et al. [112]
Water-in-oil microemulsion technique	8		Zinc	Iron oxide	Histidine-tagged phycobiliprotein	Chen et al. [22]
–	20		Iron	Iron oxide	Phosvitin phosphopeptides	Zhang et al. [125]
–	10		–	Fe_3_O_4_–Na_2_Ti_3_O_7_	Albumin and haemoglobin	Zhou et al. [131]
Coprecipitation method	14		Nickel	Cobalt ferrite	Tpv-sHSP 14.3	Aygar et al. [4]
Hydrothermal method	30		Nickel	Iron oxide	Histidine-tagged proteins	Cao et al. [15]
Coprecipitation method	11		Co^2+^	Iron oxide	Benzaldehyde lyase	Tural et al. [108]
–	35		Nickel	Iron oxide	Histidine-tagged proteins	Rashid et al. [93]
Coprecipitation method	30		Nickel	NiFe_2_O_4_	Histidine-tagged proteins	Mirahmadi-Zare et al. [76]
Water-in-oil microemulsion technique	200		Co^2+^	Iron oxide	Histidine-tagged proteins	Feng et al. [38]
Solvothermal Method	225		Gadolinium	Iron oxide	Phosphopeptides	Jiang et al. [57]
–	36		Zirconium, Nickel and Copper	Iron oxide	Histidine-tagged green fluorescent protein	Fraga García et al. [42]
Coprecipitation Method	75		Nickel	Iron oxide	Histidine-tagged enzyme	Wang et al. [113]
–	9		Nickel	Iron oxide	Histidine-tagged fluorescent DsRed proteins	Bloemen et al. [8]
Coprecipitation Method	75		Nickel	MnFe_2_O_4_	Histidine-tagged proteins	Rashid et al. [94]
Modified polyol method	68		Nickel	Iron oxide	Single-domain antibody ToxA5.1	Parisien et al. [85]
–	16		Zirconium	Iron oxide	α- and β-caseins	Zhao et al. [130]
–	27–31		Copper	Iron oxide	Bovine haemoglobin and bovine serum albumin	Zhang et al. [129]
One-pot hydrothermal method	83		Nickel	Graphene	Poly-histidine-tagged proteins	Liu et al. [68]
Solvothermal method	281		Copper	CoFe_2_O_4_	Haemoglobin	Liu et al. [70]

**Table 3:** Synthesized and natural polymers as functionalizing agents of MNPs in protein purification

Method of synthesis	MNP size (nm)		Chelating agent	Core material	Target protein	References
Miniemulsion/solvent evaporation technique	105		Ni^2+^-NTA	Iron oxide	Hexa-histidine-tag green fluorescent protein	Jose et al. [60]
Miniemulsion polymerization	11		P(NIPAM-co-AA)	Magnetic poly(methyl methacrylate)	IgG	Borlido et al. [10]
	215		CMPEI-Ni^2+^	Iron oxide	Histidine-tagged peptides	Chang et al. [19]
Co-precipitation method	50–100		Chitosan	Iron oxide	BSA	Gonzalez et al. [48]
–	98		Polyethyleneimine	Iron oxide	Phosphopeptides	Chen et al. [22]
–	10		NTA-Ni^2+^	Iron oxide	Histidine-tagged peptides	Guo et al. [50]
	10		Carboxymethyl cellulose and chitosan	Iron oxide	Peroxidase enzyme	Kurt et al. [63]
Co-precipitation method	15		Carboxymethyl chitosan	Iron oxide	Lysozyme	Sun et al. [104]
	30		Cibacron Blue F3GA	Iron oxide	BSA	Amiri et al. [2]
	10		Carboxymethyl chitosan	Iron oxide	BSA	Sadeghi et al. [95]
Solvothermal method	20		Polydopamine	Iron oxide	Hydrophobins	Cordova et al. [27]
	–		mPEG	Iron oxide	IgG and BSA	Cheng et al. [23]
Reversible addition − fragmentation chain-transfer polymerization	1000–3000		poly(*N*-isopropylacrylamide)	Iron oxide	Lactoferrin, BSA, and lysozyme	Paulus et al. [87]
Surfactant free emulsion polymerization	163		*N*-acetyl-d-galactosamine	poly(HEMA-Man-OPA)	Lectin	Feyzioğlu Demir et al. [40]
Co-precipitation method	200		Chitosan	Iron oxide	IgG	Kavas et al. (2012)
RAFT polymerization	10		10 kDa pNIPAm	Iron oxide	Elastin-like polypeptide receptors	Ta et al. [105]
–	79		Poly(styrene-sulfonate-*N*-isopropylacrylamide)	Iron oxide	Lysozyme	Zhang et al. [126]

### Antigens and antibodies

Antibodies and antigens present in biological samples are analysed by immunoassays. Once the immunoassays are combined with MNPs, it becomes easier to separate the analytes from the other components in the reaction medium. The combined materials have been applied in the food, environment, and health sectors [89]. For instance, diseases linked to their antigens and antibodies are more easily detected, and it is now possible to extract higher yields of exosomes with the MNPs-immunoassay technique, which is not the case with the single use of any of the well-established old methods. To diagnose cancers, exosomes play important roles. However, their separation is difficult due to their trace volume in the blood. Recently, biotin-labelled MNPs complexed with magnetic nanowires were developed for multifunctional purposes, which resulted in the exertion of stronger magneticity on the MNPs, and the subsequent increase in separation speed and efficacy [67]. This biotin label fostered stronger nanowire-antibody bonds. The antibodies studied include those that mainly target exosomes like the antagonists of CD9, CD63, and CD81 for exosome extraction and quantification. The nanowires were elongated thus providing large surface areas that allow the reachable antibody-coated surfaces to capture exosomes [67]. The resultant effect of this is an increased sensitivity as well as higher yields of well-purified exosomes when compared to conventional techniques.

It is quite important to note that poor diffusion affects some MNPs which can lead to a smaller surface area with regard to the volume of antibodies or an increased length of incubation time, both of which reduce the total number of antibodies capable of binding to the MNPs’ surface, hence, blurring the recognition of protein and the efficacy of the process. However, a heat-responsive micro-nano binary system has been developed comprising of MNPs [poly *N*-isopropylacrylamide (*p*NIPAM)] and antibody (Ab), which are the separation component and recognition component, respectively [79]. They are easily detached from each other once the temperature is increased, and the reagent in the system specifically targets immunopositivity for HIV core protein, p24. Within two minutes, p24 could be captured in the serum based on the binding of *p*NIPAM to the polymer-Ab complex. The efficiencies of only Ab or polymer-Ab complex are ten and five times lower, respectively than that of the binary system.

From another interesting viewpoint of improving sensitivity and efficacy by coupling or complexation of antibodies with MNPs, Xu et al. [120] developed a bio-membrane-coated single-domain nanocrystal with magnetotactic bacteria. This complex is also referred to as bacterial magnetic nanoparticles (BMPs) or magnetosomes. In their study, the magnetosomes were further complexed with protein A (ΔF-BMP-FA) before binding it to mammalian antibodies. This resultant complex with an antibody (ΔF-BMP-FA/Ab) could detect the *Vibrio parahaemolyticus* pathogen in an immunomagnetic assay. The complexation involving BMP-A-Ab led to a 90% maximal rate of the pathogen capture. This method is regarded as a green synthetic approach because living organisms are employed, leaving little or no room for toxicity issues or concerns.

Super particles like iron oxides have also been used to functionalize MNPs to increase the speed and sensitivity of immunoglobulin (IgG) assay [111]. The iron oxide MNPs were coated with silica to form the nanocomposites (Fe_3_O_4_@SiO_2_) that could bind human IgG when exposed to immobilized goat-anti-human IgG. Unlike the ΔF-BMP-FA/Ab functional complex, these nanocomposites have no biosynthetic components involved. Gold silicon-coated anti-human IgG or magnets could also be used in isolating the composites [111]. The studies explored so far indeed contributed to the use of MNPs and their nanocomposites both in living and non-biological systems to improve the separation and purification of antibodies and antibody-bound exosomes associated with cancers.

### MNPs-functionalized biomolecules

Functionalized MNPs with ligands like biomolecules increase the recognition and binding of proteins, resulting in magnetic separation and purification. This is illustrated in Fig. 1. Antibodies, peptides, proteins, glycoproteins, DNAs, and amino acids are among the biomolecules that are separated, purified, and characterized based on the magnetic characteristics of the core nanocomposites of MNPs.

Glycoproteins, for example, are important post-translationally modified proteins via the process of glycosylation. They are particularly the target of nanocomposite-based diagnoses of cancer and neurodegenerative diseases [39, 117]. After enrichment with biomolecules, glycopeptides could be identified by using MS to analyse the patterns of glycosylation [69]. This was carried out in the study of Wu and colleagues [117] who enriched human seral glycopeptides with newly developed zwitterionic hydrophilic polydopamine-coated magnetic graphene composite (magG/PDA/Au/L-Cys). The analytical outcome of the enriched complexes by MALDI-TOF–MS showed that they are highly sensitive, selective, hydrophobic, biocompatible, and reusable.

Biotechnologists find it challenging to separate, purify and extract tagged and recombinant proteins, bacterial proteins and peptides, myosins, proteases, intracellular proteins, lectins, mRNAs, antibodies and several other types of proteins like proteins A and G. Nevertheless, functionalized biomolecules-MNPs composites offer great opportunities and alternatives about their superparamagnetic, modifiable surface and high adsorption properties, making them great facilitators of separation and recovery from various media matrices and fields. They are more efficient and cost-effective as compared to, for example, protein A or G affinity chromatography methods. MNPs coated with affinity-based separation systems now have the potential to improve the demerits of chromatography. Recently, a composite synthesized from protein A and magnetic SiO_2_ microspheres, denoted as Mag(SiO_2_) was successfully developed to purify IgG, even at a low concentration as < 50 mg/mL, and could isolate rabbit seral IgG at a purity rate above 95% [97]. The magnetic field was intact without any tangible protein A leaching observed. This is not the only example, functionalized MNPs have been complexed with protein A and chlorophenylsilane to form CPTMS@MNPs nanocomposites that could purify antibodies on a commercial scale [61]. The researchers could orientate the surface of CPTMS@MNPs to enhance its ability to bind and increase protein and antibody immobilization by 11.5- and 7-folds, respectively in comparison with conventional MNPs that are commercially available as “Dynalbeads”. Protein A and G affinity separation and purification have received a lot of attention. In another study, gold-coated protein A and G nanoconjugates were designed to extract human IgG from cell culture supernatants and human plasma [69]. These functionalized MNPs efficiently extracted the targeted antibody from the media matrix, serving as an excellent option to Dynabeads that operate based on the immunoaffinity trap of magnetic beads.

For myosins, Chen and colleagues [21] purposed to purify myosin subfragment-1 using a synthesized DNA-polyoxovanadate biocomposite attached to streptavidin-coated MNPs (V12O32-DNA@SVM) and observed that as little as 0.1 mg of the nanocomposite could efficiently absorb the myosin subfragment-1 based on the intensive binding at the ATP-V12O32 junction. This purification technique had an efficiency rate of 99.4% and serves as the backdrop for further application in myosin extraction from porcine crude protein. This appears easy enough but for sialoglycoproteins, it is a bit challenging because of their rarity and low availability in biological systems. Nonetheless, an attempted study by Du and co-workers [30] showed some potential. The authors ligated disulfide-linked amino-oxy-functionalized MNPs with periodate-coated glycoproteins before separation in a magnetic field of Fe_3_O_4_. The sialoglycoprotein was released by reducing agents from the terminal glycan chains and was analyzed using sodium dodecyl sulfate–polyacrylamide gel electrophoresis and tandem mass spectrometry. The findings summarily described this technique as a great tool for the selective separation of sialoglycoproteins.

Microbial protein analysis and enrichment are also carried out with MNPs which may be first exposed to biomolecular functionalization and then complemented with MALDI-TOF mass spectrometry. For instance, a magnetic nanoparticle modified with 3-aminopropyltriethoxysilane and covalently linked to the surface of β-lactam antibiotic amoxicillin was developed by a group of researchers [52]. *Escherichia coli* and *Staphylococcus aureus* were modelled as the target of β-lactam affinity-binding of the β-lactam-coated MNPs. It was observed that the magnetic particles could easily separate the bacteria. The molecular weight of penicillin-binding proteins of the *E. coli* based on MALDI-MS was within the range of 20–55 kDa while that of *S. aureus* was 49.570 kDa. The success of this method indicates that rapid and selective bacterial detection in the diagnosis of bacterial infections with MNPs is attainable, employing simplistic, reusable, cheap, non-toxic, timely, and biocompatible materials. More reports whereby biomolecules complexed with functionalized MNPs potentiate solutions to the challenges of separation and purification in the biotechnology industry are described in Table 1.

### Ionic metals

A trendy purification technique nowadays is known as immobilized metal ion affinity chromatography also known as IMAC. This method targets poly-histidine peptide pre-tagged enzymes and proteins. The affinity of poly-histidine peptides towards ionic metals like calcium, iron, zinc, copper, nickel, cobalt, or gallium could be engaged and well utilized in the process of protein binding. A major application of this approach could be found with the recombinant proteins, as they are obtained in their purest state through a C- or N-terminally fused affinity tag, most especially using the histidine tag [102]. Once this is achieved, the structure and function of the recombinant proteins could be improved. An affinity column is required for the purification of proteins, and if it is absent, histidine-tagged proteins which usually have ≥ 6 histidine residues in sequence could be bound to the target protein before their purification through any other appropriate column. MNPs functionalized with the mentioned metals indeed portend excellence when histidine-tagged proteins are to be separated and purified. Figure 2 illustrates this rationale.

Iron oxide and gold MNPs are increasingly used in separation and purification procedures, analytical chemistry, and many biological experiments because they are chemically stable and biocompatible. A recent investigation involving the complexation of iron oxide and gold core–shell with nickel showed their potential use in protein purification [51]. The researchers prepared the sample with Fe_3_O_4_@Au/NTA-Ni^2+^ and found that the functionalized MNPs yielded ≥ 96% efficiency rate without losing chelation. The high surface area of the MNPs has contributed to the success of this experiment, even though they have a large nanostructure.

Nickel is also popular as one of the ionic metals used with IMAC. Many core/shell MNPs could be functionalized with nickel to purify histidine-tagged proteins. A nickel column was developed and used synergistically with histidine tags to separate β-glucosidase and was found to be very effective at obtaining the highest purity yield, thermal stability, and binding rate ever, much more than using only nickel column [11]. However, it is not cheap and cannot be reused. Therefore, simpler and cheaper methods of purifying histidine affinity-tagged proteins must be developed. To this end, carboxylmethylaspartate (Co^2+^-CMA) and nickel nitrilotriacetic acid (Ni^2+^-NTA) were complexed with resin for commercial applications based on safe coordination of the cobalt and nickel metals with the histidine residues, and subsequent regeneration and reusability with regard to the metal-binding effect and stability of the nanocomposites [11].

Indeed, it is difficult to substitute nickel for IMAC use. Nonetheless, many attempts to do so with other ionic metals have been ongoing for years. For instance, in a bid to separate lysozyme, Fe_3_O_4_@SiO_2_-IDA microspheres were developed and bound to Zn^2+^, Cu^2+^, and Ni^2+^ prior to evaluation using the histidine-tagged protein system [81]. The researchers reported that adsorption of the histidine-tagged protein by the metals was possible but not their desorption because Cu^2+^ ions have ≤ 15% desorption rate as compared to others i.e., zinc and nickel ions. So, Fe_3_O_4_@SiO_2_-IDA-Ni^2+^ was examined and found to demonstrate > 97% purity and can be reused. This is quite related to the significance of nickel ion’s adsorption, desorption, and biocompatibility features. However, the chelation of the nanostructure to the protein in question and the pH of the process medium are key parameters under IMAC procedures because the higher the chelating capacity, the cheaper the cost of purifying the sample would be as fewer nanomaterials would be required. A group of researchers used ~ 200 mg/g carboxymethyl chitosan MNPs (Fe_3_O_4_@PEG-CM–CTS) with Fe^3+^, Cu^2+^, or Zn^2+^ ionic metals for lysozyme adsorption [104]. They observed that Cu^2+^-coated MNPs thrive in performance at lower pH while the MNPs treated with Fe^3+^ and Zn^2+^ performed best at a higher pH. In addition to this, the particle size was connected to the activity of the MNPs, as the smaller size of 15 nm greatly affected their chelating capacity.

Phosphopeptides have also been separated and purified by IMAC. The complexation of pyridoxal 5-phosphate with Fe_3_O_4_@SiO_2_ and certain ionic metals like Ga^3+^, Fe^3+^, and Ti^4+^ to separate and purify phosphopeptides led to some amazing results [112]. It was observed that the composites ligated with Ti^4+^ effectively aided the purification of phosphopeptides ≥ 84%. They were also selectively separated from human serum, rat brain fragments and digested components of defatted milk. The other ionic metal complexes did not perform so well. However, the results obtained compete well with the commercially available purification techniques of phosphopeptides. Phosphopeptides are not the only tested biomolecules, glycopeptides have been studied using IMAC separation and purification, and they mostly involve organic components with ionic metals. Biological samples with glycoproteins treated with the organic-ionic metal complex yield well-separated and purified *N*-linked glycopeptides (≥ 83% purity) thanks to their lipophobicity and high surface area [118], [73]. More examples of studies that attempted or successfully separated and purified glycopeptides and Phosphopeptides from different biological samples are presented in Table 2.

Haemoglobin (Hb) is another protein molecule as well as a complex iron compound that could be separated and purified with ionic metal-complexed MNPs. In a study, Cu^2+^-EDTA-Fe_3_O_4_ nanocomposites were demonstrated to possess a strong chelating property except in basic pH [29]. The researchers found that the ionic concentration, strength, and contact timeline significantly improved the adsorptive effect of the nanocomposites, factoring along other IMAC parameters. However, the lower desorption reported on the copper-coated composite is the downside of this study. The purification of protein C with Cu^2+^, Zn^2+^, and Ni^2+^ as functionalizing metals complexed with magnetic poly(hydroxyethylmethacrylate-*N*-methacryloyl-(L)-histidine) nanoparticles under the variables of ionic strength, pH, concentration, temperature and time showed an overall separation efficiency and purity greater than 93% [25]. The most potent of the studied composites are nickel-coated MNPs with 73 nm size, proven to be reusable for protein C purification. Multifarious studies (Table 2) linked ionic metals as functionalizing agents of nanocomposites, especially with the use of IMAC, to great purification efficiency and reusability potentials. Therefore, IMAC is very useful but with its limitations as well. Materials used in the system’s separating chamber like solution and metals could be exhaustive, but still, more ionic metals are offering options for nickel dominance. The other parameters like contact or adsorption time, ionic strength, and pH also pose limitations to the IMAC system because they require optimization.

### Polymers

Metals have gained increasing popularity with the MNPs possibly due to their electric charges that enhance easy adsorption and chelation to their targets. In the same vein, polymers offer a wide range of opportunities for MNPs functionalization and they have been applied in the science of chemical catalysts and biomedicine [33, 35, 106]. Based on the available literature, the commonest polymer used to enrich MNPs is iron oxide. For instance, iron oxide nanocomposite (Fe_3_O_4_@MPS@PMAC) synthesized from γ-methacryloxypropyltrimethoxysilane (MPS) and poly[2-(methacryloyloxy)ethyl]trimethylammonium chloride (PMAC) was modified on the surface with lipophobic liquid ions to separate and purify glycopeptides [59]. The researchers found the composite to be hydrophilic and effective as it possessed an excellent binding power (100 mg/g) and a high purity recovery rate (≥ 80%).

Exosomes are a group of membrane-bound extracellular vesicles produced in eukaryotes’ endosomes and are thus, important biomarkers of cancer prognosis. They must be isolated from other biomolecules to obtain testing accuracy. In this regard, iron oxide MNPs complexed with polyethylene glycol (PEG) were designed to isolate exosomes by removing other free proteins in the tested biological sample [18]. The authors introduced temperature and concentration as variables of the method during which isolation efficiency increased when the concentration was raised by 3% at room temperature. The PEG-iron oxide nanocomposite showed better purification performance in terms of speed and simplicity than the most common conventional method of exosome purification i.e., differential centrifugation. Polymers are multifarious and versatile in nature,these are qualities quite important for binding to MNPs to enhance separation and purification. Another group of researchers also used PEG complexed with starch-coated MNPs to isolate IgG monoclonal antibodies via the steric exclusion principle whereby just as little as 1.3% of an embedded nano mass of IgG could adequately enable an efficient magnetic collection of the associated IgG at a 98% purity rate [45]. The study outcome and many others show that polymer-complexed MNPs offer versatility and numerous potentials for separating and purifying biomolecules like proteins and antibodies. Figure 3 has been constructed to illustrate this point.

It is not just nanoparticles that could be useful in the design of magnetic particles-functionalized separation and purification procedures for proteins, microparticles could also compare well, even though not as much as the former based on surface area differences. Some studies have explored microstructures to separate peptides, proteins, and glycopeptides. An example is the development of hydrophilic particle-coated plates (CP) and C18 particle-coated PDMS microplates (C18MPs) to purify peptides, proteins, and glycopeptides [66]. The methods were successful, simple, and rapid, with CP attaining superior efficiency than C18MP and both achieving 64% purity. This performance is lower than that attainable by nanocomposites, a reminder of the role of surface area in the design of MNPs-functionalized purification systems. Another group of researchers employed carbon-based hollow microspheres to separate peptides by embedding iron oxide MNPs in the microspheres [24]. The complex was applied to protein fragments, biosamples, and standard mixtures, and showed better performance than the commercial ZipTip C18 magnetic silica used in peptide separation. In this case, the microstructure possessed some degree of hydrophobicity, and because they are mesoporous, they have a high surface area capable of adsorbing peptides at very low concentrations, with an overall performance similar to that of nanostructures. This is a very effective tool that breaks through the odds of nanocomposite-based magnetic separation techniques.

It is noteworthy that any MNPs-designed systems must be biocompatible, having no toxicity or harmful effects. They should also be able to respond to stimuli if possible. Two studies have recently demonstrated these criteria [47, 56]. Specifically, Ghanbari Adivi and colleagues [47] used ultrafine agarose, a natural biopolymer known to be biocompatible, to coat superparamagnetic iron oxide nanoparticles (AC-SPIONs), epoxy-activated by epichlorohydrin and aminated by ammonium hydroxide in a one-step method to purify gallic and ellagic acids. The results obtained showed that the functionalized AC-SPIONs possess great adsorptive and in vitro drug release properties in phosphate-buffered saline at a pH of 7.4. The results indicate AC-SPIONs as applicable in magnetic solid-phase extraction, drug delivery, protein purification, and enzyme immobilization techniques [47]. The other authors [56] used a diblock copolymer of poly(acrylic acid)-block-poly(*N*-isopropylacrylamide) (*p*AAc-b-*p*NIPAAm) to complex iron cations (Fe^2+^/Fe^3+^) in water followed by adding NH_4_OH to induce the formation of the iron oxide particles. Without any surface modifications, the synthesized MNPs responded to temperature stimulus in one step and were stable with reported purity efficiency > 95%. The researchers further demonstrated the practical use of the stimuli-responsive MNPs by incorporating them into a temperature-responsive binary reagent system whereby they not only separated mouse IgG (a model protein biomarker) but also purified human-derived extracellular vesicle. The study serves as a backbone for exploring stimuli-responsive polymers for separation and purification methods applicable in protein science, biomedicine, and other areas of research.

Table 3 presents some studies that have shown that natural and synthetic polymers are very useful in the design of functionalized MNPs targeting the purification of protein-related biomolecules. Enzymes have also been separated and purified by polymer-coated MNPs as they offer more speed and cost-effectiveness. For example, a one-step purification procedure created by immobilizing azocasein onto iron oxide MNPs and complexed with polyaniline was applied to purify proteases between 25 and 65 degrees Celsius and at 5–11 pH range [80]. The purification efficiency was greatly enhanced more than 55 times when compared with the conventional chromatographic method of protease purification. The protease activity remained stable at 45 °C and was optimum at the pH of 9, indicative of safe storage at room or wider ranges of temperature and pH. All these studies demonstrate that polymers are excellent MNP-core functionalizing agents. They are cheap, ubiquitous, and versatile in composition, structure, and function, making them suitable for multifarious purification and biological applications, even on a commercial scale.

### Graphene nanoparticles

The mechanical, thermal stability, electrical, and chemical properties of graphene make it a special carbon-based material used in a wide variety of industries and sectors. These include biosensors, teeth and bone-restoring agents, imaging devices, chemotherapeutic and drug delivery agents, and various electrical devices [31, 32, 34, 86, 91]. Certain reports indicated that graphene and its oxide could recognize, separate, and purify proteins, peptides, and phosphopeptides once its surface has been modified. For example, the surface of graphene was modified by coating it with titania (TiO_2_) to capture phosphopeptides from complex biological samples [24]. The researchers composited graphene scaffolds with Fe_3_O_4_ nanoparticles to actuate and fully cover the porous titania nanostructures as affinity coating. It was observed that the affinity graphene nanocomposites selectively captured and rapidly separated low-abundant phosphopeptides from complex biological samples [24]. Graphene becomes more selective and biocompatible once its surface is modified, and its structural linking to Fe_3_O_4_ enhances its smooth removal from the biological samples. This is applicable to water treatment.

Some other studies reportedly employ multi-walled nanotubes (MWNTs) or graphene oxide (GO) for the purpose of enhancing the separation and purification processes. The MWNTs are carbon-based and act to support the molecular imprinted polymer (MIP) for selective recognition of bovine serum albumin (BSA) [129]. The Fe_3_O_4_ nanoparticles were complexed with MWNTs-MIP using methacrylic monomer to form MWNTs@Fe_3_O_4_-MIPs, which could selectively adsorb BSA at 52.8 mg/g. On the other hand, when GO was composited with Fe_3_O_4_ with the aid of acrylamide monomers as the co-polymerizing agents and a cross-linking agent, divinyl benzene, the toxic heptapeptides known as microcystins (produced by freshwater bacteria) were easily recognized, and separated [84]. This nanocomposite also derived its solid backing from MIP to form a T-MMIP hybrid, which bound to the microcystins YR, LR, RR, WR, LW, LY, LA, and LF, and separated them on the basis of its magnetic nanocomponents. These studies have one thing in common, the MIP. The MIP is known for its selectivity and separation capacity based on its specific polymeric template binding. The challenges with MIP protein recognition are associated with the conformation, solubility, and molecularity of the protein targets. Thus, surface molecular imprinting involving a thin polydopamine layer imprinted and developed by polymerizing dopamine onto Fe_3_O_4_-GO nanoparticles was designed [20]. The nanocomposite could bind and remove BSA from biological samples due to its large surface area. More than 117 mg/g BSA was adsorbed from a mixture of ovalbumin, lysozyme, ribonuclease A, and Hb, while the composite maintained stability throughout the adsorption and desorption processes, without losing its binding ability.

Lastly, carbon nanotubes were employed by another group of researchers to separate proteins based on their size differences [26]. The study is illustrated in Fig. 4. The authors grafted thermal-sensitive *p*NIPAM and Fe_3_O_4_ nanoparticles onto the carbon nanotubes’ open ends of 15 nm diameter by using the surface-initiated atom transfer radical polymerization (SI-ATRP) method. The cavities of the nanotubes could transport both proteins and ions effectively and could be reversed in operation in a magnetic-thermal sensitive way, thereby separating various-sized proteins. Graphene MNPs-based separation techniques like this contribute greatly to the scientific advancements and applications of novel technologies to biosensors, purification filters, biofilters, and artificial cells.

### Other functionalizing agents

Several non-specific nano-agents have been employed to develop affordable complexes that aid rapid and smart separation and purification of peptides, chromatin, enzymes, glycopeptides and proteins. Zhang and colleagues [127] employed a three-sited glycosylated *N*-glycoprotein also known as asialofetuin as a model when fabricating amine-functionalized magnetic nanoparticles by a one-pot method to selectively enrich glycopeptides and phosphopeptides. The complex mixture of peptides, phosphopeptides and glycopeptides at a 10:1:1 ratio was analysed, and the recovery rates obtained for glycopeptides and phosphopeptides were 76% and 88%, respectively. This was a simple, easy-to-use, affordable and fast procedure, shown to be highly selective and efficient. Therefore, it is proven once again that magnetic MNPs (particularly Fe_3_O_4_ nanoparticles) are crucial in this field of research. They are cheap, fast, simple, easy to use, biocompatible, and can be renewed. These iron oxide particles have been used with a *p*-aminophenol affinity ligand to isolate and purify the α-amylase enzyme from bovine milk [36]. The ligand used was immobilized by linkers of various lengths, and when longer ones were used, greater separation efficiencies were attained, as high as ≥ 48-fold purification efficiency and a recovery rate of 49.66%.

Superparamagnetic nanoparticles have been used in chromatin and protease chelation and release too. The mammalian chromatin was separated from lysed cells by employing salicylic acid-coated magnetic nanoparticles (SAMNPs) in mammalian culture [132]. It was a simple, easy, and sustainable technique that has several advantages over the conventional procedures of extracting chromatin such as centrifugation. The fixing and fragmentation steps in the old technique could create structural errors and distortions in the mammalian chromatin. In addition, the magnetic particles were utilized to design a nano-hydro system to purify protease using the concept of *click chemistry* [100]. It is regarded as a well-dispersed, magnetic, and cleavable copper-free click-nano-reagent in which an azide-functionalized dye could bind to the iron oxide particle, and desorb with 13 mM fluoride solution without losing the protease activity.

## Large-scale application of MNPs, recovery, and challenges

Small MNPs with a narrow size distribution are of great interest for several biomedical applications. When the size of the particles decreases, the magnetic moment of the particles decreases, leading to a significant increase in the separation time by several orders of magnitude [116]. Most nanoparticles are smaller than 1 µm in diameter and are typically 1–100 nm in scale. An example is the Cobalt nanoparticle with graphene shell which is 5 nm in size [49]. The magnetic nanoparticle clusters that are composed of a number of individual MNPs are known as magnetic nanobeads with a diameter of 50–200 nm. This information coupled with industrial protocols for the use of these MNPs makes it easy to scale them up for large-scale applications. However, many of the MNPs are toxic and not biocompatible despite their high levels of magneticity. MNPs are in their early developmental stage despite being used in the separation of amino acid isomers, when compared to agelong and well-established techniques such as chromatography [22], [107]. Reusability is another major challenge of MNPs as there is a dearth of information with regard to this. One of the popular currently available MNPs commercially is dynabeads. They are superparamagnetic spherical polymer particles with a uniform size and a consistent, defined surface for the adsorption or coupling of various biomolecules including proteins. Yet, they are not reusable. In addition to this, a major aim of inventing MNPs is to explore simpler and cheaper means of separation and purification. This aim has often been defeated and large-scale intervention in the marketplace has not reduced the cost of purchase.

Other than the challenges mentioned and that of large-scale optimization, the recovery of MNPs is also crucial. In this regard, the magnetic nanoparticle recovery device (MagNERD) is potentiates the effective solution. Powell et al. [90] optimized a magnetic nanoparticle recovery device (MagNERD) to separate, capture, and reuse superparamagnetic Fe_3_O_4_ from treated water under continuous flow conditions. The MagNERD’s efficiency in recovering nanoparticles depends on reactor configuration, including the integration of stainless-steel wool around permanent magnets, hydraulic flow conditions, and MNP uptake. The researchers found MagNERD to efficiently remove Fe_3_O_4_ in the form of a nanopowder, up to > 95% at high concentrations (500 ppm), under scalable and process-relevant flow rates (1 L/min through a 1.11-L MagNERD reactor), and in varying water matrices including ultrapure water and brackish water.

The award-winning ThermoFisher developed CTS DynaCellect magnetic separation and recovery system consists of an intuitive, programmable interface, integrated magnet-rocker, and fluidics panel that is capable of exceptional cell recovery, a wide range of reaction volumes, optimal efficiency, and a cell purity rate > 95% while maintaining cell viability. The magnetic recovery system is designed to easily scale from development to clinical and commercial manufacturing of biosamples. Regardless, the sterile consumables in the flexible process are single-use, while the provided software is subjected to upgrade. Therefore, more studies exploring the possibility of large-scale application of MNPs capable of overcoming these limitations are warranted.

## Future outlook and conclusions

To employ cheap, rapid, and simple protocols of separating and purifying proteins in all forms, magnetic nanoparticles would play critical roles that conventional methods could not offer. Several inorganic and organic compounds and molecules could be functionalized with MNPs to enhance the isolation, separation, and purification of peptides and proteins from multifarious sources, calling for more validatory studies incorporating in-depth analyses and applications. The MNPs could be reused and efficaciously manipulated with various nanomaterials such as antibodies, biomolecules, ionic metals, graphene or carbon-based compounds, polymers, and metal oxides, leading to highly improved efficiency, adsorption, desorption, and purity rate. It is important to factor in parameters such as charge, size, core–shell, lipophilicity, lipophobicity, and surface energy of the MNPs when considering protein selectivity, chelation, separation, and purity.

## Data Availability

The raw/processed data required to reproduce these findings cannot be shared at this time due to technical or time limitations.
